# Institutional Notes and News

**Published:** 1909-11-13

**Authors:** 


### Institutional Notes and News.

### JUBILEE OF THE NATIONAL HOSPITAL.

Last week was a notable one in the history of the National Hospital for the Paralysed and Epileptic. On Thursday, November 4, the King opened the Jubilee Extension buildings, and on Tuesday afternoon, November 2, the Duchess of Albany received, on behalf of the Jubilee Fund, a number of purses containing altogether about £350. Many of the amounts in the purses were collected by patients who presented them personally to the Duchess. Prior to this function there was a thanksgiving service in the chapel of the hospital to commemorate the 50th anniversary of the institution, which was founded on November 2, 1859, which the Duchess of Albany attended. The sermon was preached by the Archbishop of Canterbury.

### ROYAL HOSPITAL FOR INCURABLES.

The one hundred and eleventh election for the benefit of the Royal Hospital for Incurables, Putney Heath, will be held at the Cannon Street Hotel on November 26. Thirty candidates will be elected. The election will be presided over by Mr. H. J. Allcroft, the treasurer of the institution.

### EASTBOURNE CONSUMPTIVES.

The Eastbourne Ladies' Visiting Committee urge the necessity of removing all consumptives from the workhouse, and placing them in a separate building arranged on sanatorium principles. The Clerk has been instructed to write to the several Boards of Guardians in East Sussex, asking whether they would co-operate with the Board in forming a joint committee for the purpose of providing and maintaining an institution for the reception and treatment of persons chargeable to the rates suffering from phthisis, and whether they would be prepared to appoint delegates to attend a conference on the subject, which conference, the committee suggests, should be held at Lewes.

### PORTSMOUTH ISOLATION HOSPITAL.

The Health Committee of the Portsmouth Corporation has decided upon an addition to the Infectious Diseases Hospital. At present when a child who has been suffering from scarlet fever or other infectious disease is sent home convalescent the father, if, as happens in many cases, he is employed in the Dockyard, is debarred from returning to his work for several days until all risk of carrying infection has passed away. This is a serious matter for the family, and to obviate it the Health Committee has resolved to build a convalescent ward, where the children may be kept as long as may be necessary. The building will not cost more than about 500*l.*, and the Finance Committee, recognising the urgency of the proposal, has agreed to the payment of this sum out of revenue.

### BANGOR HOSPITAL.

At the last monthly meeting of this hospital the Treasurer's report showed a credit balance of £47 0s. 11d. In connection with the new hospital, the foundation stone of which will probably be laid within a month, the following were appointed to act as a building committee: Mr. M'Meekan, Miss Connor, and Dr. Moore. On the motion of Mr. Mussen, seconded by Mr. Matthews, it was resolved that the Committee ask Miss Connor to perform the ceremony of laying the foundation stone.

### BARRY HOSPITAL.

The Hospital Committee has recommended that the Council appoint the medical staff for the hospital, and that a special committee be appointed to prepare a scheme for this purpose and report whether this work could not be carried out in conjunction with the other medical works under the Council. The British Medical Association suggested a scheme of rotation on the staff of the hospital for all the doctors of the town, working it in three monthly periods, the payment to each doctor to be £20. The Hospital Committee has now been instructed by the District Council at its last meeting to prepare a scheme in accordance with a previous resolution.

### A POINT OF LAW.

The governors and secretary of the Royal Berks Hospital had the somewhat doubtful privilege some time ago of helping the lawyers to define what the law means by "a public institution." The point was whether a hospital's laundry were a factory or workshop within the meaning of the Acts of 1901 and 1907. If it is, then H.M.'s inspector of factories has to be admitted. But his admission at the above hospital was objected to. It was decided, however, that this was a technical offence, for that the Royal Berks Hospital was in the meaning of the Act of 1907, a public institution.

### PORTSMOUTH HOSPITAL'S FINANCES.

The report of the Finance Committee of the Royal Portsmouth Hospital presented at the October monthly meeting showed a decrease of income and an increase of overdraft. Mr. E. H. Cooper, however, who presented it, said that they did not have to pay interest on the overdraft.

### MANCHESTER ROYAL INFIRMARY.

Mr. Charles Hopkinson, chairman of the Building Committee, was entertained at dinner at the Queen's Hotel, Manchester, on October 22 by the Board of Management and the Medical Board of the Manchester Royal Infirmary. Sir William Cobbett, the chairman of the Royal Infirmary, in proposing the toast of "Mr. Charles Hopkinson," said that from the date of the death of the late chairman in 1904, who was also chairman of the Building Committee, no detail of the construction of the infirmary had been too minute for Hopkinson's attention, and that it was largely due to him that the buildings had been erected in three and a half years from the letting of the contract. In his reply Mr. Hopkinson remarked that he had set before himself the three aims of efficiency, sound economy, and expedition, and passed words of praise on the work of the architects, Messrs. Edwin T. Hall and John Brooke. Other toasts followed, and a memorable occasion in the history of the most recent of great hospitals was with them happily concluded.

### DUDLEY GUEST HOSPITAL.

At the annual general meeting of the Dudley Dispensary feeling reference was made to the death of one of the trustees, the late Mr. J. H. Smith, who lost his life a few months ago in such a tragic and sad way. He had been a good friend to that institution for a great number of years. He had been one of the trustees for 18 years, and his loss will be severely felt by the institution.

